# Dominant *Mycobacterium tuberculosis* Lineages in Elderly Patients Born in Norway

**DOI:** 10.1371/journal.pone.0008373

**Published:** 2009-12-18

**Authors:** Wibeke Kinander, Torbjørn Bruvik, Ulf R. Dahle

**Affiliations:** Norwegian Institute of Public Health, Oslo, Norway; MRC Laboratories, Gambia

## Abstract

**Background:**

During the previous century Norway had a high incidence of tuberculosis, but no molecular epidemiological studies could be performed and these previously epidemic strains have been disappearing during the last decades. Currently, tuberculosis among native Norwegians is in the elimination phase, and it is still not known what type of *M. tuberculosis* was so efficiently controlled during the second half of the 20th century. However, many elderly Norwegian-born people still develop TB that cannot be clustered to imported or recently transmitted strains of *M. tuberculosis*. Thus, the majority of these cases are results of reactivation of disease that was transmitted many decades ago.

**Methodology/Principal Findings:**

A total of 213 strains of *M. tuberculosis* isolated during 1998–2005, from patients born in Norway before 1950 were genotyped in the current study. The findings demonstrated a highly homogenous *M. tuberculosis* population among the patients. A total of 40% belonged to the T-family, were 35% were assigned to T1 sub- family (T2 = 0, 93%, T3 = 1, 4% and T4 = 2, 3%). As many as 35% of the isolates belonged to the Haarlem family, were 15% were assigned to Haarlem1 and 19% to Haarlem3. The remaining 25% belonged to 15 different other families. The RFLP-patterns indicated that the isolates were not a result of recent transmission, but rather represented well established strains that apparently dominated in Norway many decades ago.

**Conclusions/Significance:**

The T 1, Haarlem 1, and Haarlem 3 families of *M. tuberculosis* were abundant among patients born in Norway before 1950. The *M. tuberculosis* cases represented reactivated disease that had been acquired before 1994 and were likely to have been latent for several decades. Thus, the current study indicated that the T 1, Haarlem 1, and Haarlem 3 families may have been common in Norway, when tuberculosis represented a serious public health threat during the first half of the 20th century.

## Introduction

Studying changes in the population structure of *M. tuberculosis* is important to help understand the adaptation infectious agents undergo to control measures. Although it remains to be proven conclusively, it is conceivable that control measures such as the *Mycobacterium bovis* BCG vaccine and antituberculosis drugs have facilitated a selection of strains which are better adapted to resist such measures [1]. If so, it is important to test the efficacy of new candidate vaccines and drugs against such successful genotypes. It is equally important to identify what genotypes were controlled by those measures employed during the previous century. Preclinical experiments have clearly demonstrated that *M. tuberculosis* isolates exhibit different levels of virulence and induce various immune responses in animal models [2]–[4]. So far however, changes in the population structure of *M. tuberculosis* are largely based on studies of currently abundant strains. Few collections of isolates from the 20^th^ century are available but they may still be found in elderly patients suffering from reactivated disease.

The incidence of tuberculosis in Norway was among the highest in the world less than a century ago and currently it represents one of the lowest in the world. Tuberculosis is close to elimination among native people in Norway [5], yet we do not know what strains of *M. tuberculosis* were so efficiently controlled. The elderly Norwegian-born patients that develop reactivated tuberculosis today represent the last survivors of the disappearing Norwegian tuberculosis epidemic. The strains isolated from these patients represent a unique chance to describe a part of the *M. tuberculosis* population that was a major public health threat well into the 1960s. Such studies may represent important arguments in the common theories that given families of *M. tuberculosis* are currently emerging in various parts of the world.

## Materials and Methods

### Ethics Statement

At the Norwegian Institute of Public Health, all *M. tuberculosis* strains are routinely collected for disease surveillance. The current study is descriptive of a bacterial collection and bacterial genotypes could only be combined with the sex, year of birth, and country of birth for the patients from which the strains were isolated. Ethical approval was therefore not required. Also, the Norwegian Act relating to control of communicable diseases (http://www.lovdata.no/all/hl-19940805-055.html#7-9) obliges the Norwegian Institute of Public Health to monitor the mycobacterial population within the country on a regular basis. For these reasons, consent was not obtained from the patients to analyze the bacterial samples for this research project.

The study population comprised patients from whom at least one sample positive for *M. tuberculosis* by culture was collected during 1998–2005. The patients were born in Norway before 1950 and the *M. tuberculosis* isolates from these patients carried an IS*6110* RFLP pattern that had previously not been observed in Norway. The IS*6110* RFLP database included the patterns of all culture positive tuberculosis cases diagnosed in Norway since 1994. It was therefore considered unlikely that the patients had been recently infected within the country by imported *M. tuberculosis* strains. The species identification of the isolates was based on a 16S-rDNA hybridization technique (AccuProbe; GenProbe Inc., San Diego, USA) and standard microbiological tests (nitrate reduction and niacin accumulation tests).

A total of 1903 strains were analyzed by IS*6110* RFLP during 1998–2007. Of these were 418 isolated from native Norwegians. Strains carrying an RFLP pattern that had first been isolated in a person born after 1950 or in another country were excluded for the study. A final 213 strains were identified as likely cases of reactivated *M. tuberculosis* infection and analyzed by spoligotyping. Only 21 of the included strains carried an RFLP pattern that had also been observed in isolates from other patients. However, the 21 included outbreak strains all represented cases where the index patient was born in Norway before 1950.

The spoligotyping methodology is described elsewhere [6]. The spoligopatterns were compared by visual examination and computer assisted analyses by use of the BioNumerics, version 5.1 software (Applied Maths, Kortrijk, Belgium). The obtained spoligopatterns were assigned to families and subfamilies based on the SpolDB4 database [7] and the ‘Spotclust’ algorithm [8].

## Results

**Figure 1:**
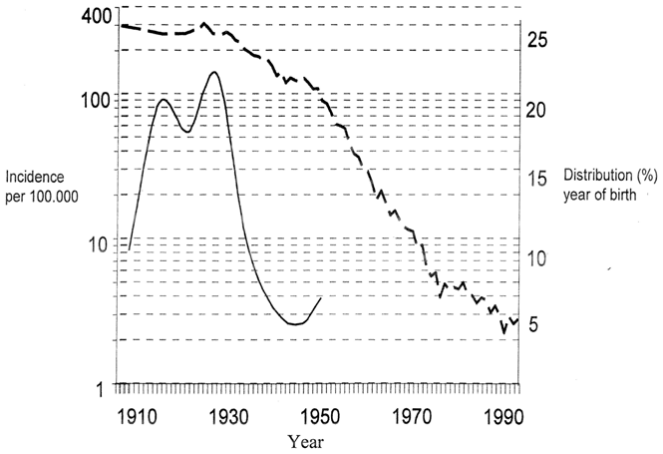
Incidence of tuberculosis and distribution of birth-year of patients from where the current *M. tuberculosis* strains originated. The incidence of pulmonary tuberculosis in Norway 1910–1990 (dotted line) is presented in a logarithmic scale (left axis). The distribution of year of birth of the patients from whom the current *M. tuberculosis* population had been isolated during 1998–2008 (solid line) is given in percent (right axis).

All included patients were born in Norway between 1910 and 1950, at a time when the incidence of pulmonary tuberculosis in Norway sunk from 400/100.000 to 100/100.000. A total of 10% of the included patients were born before 1914, 20% were born during 1915–1919, 17% were born during 1920–1924, 23% were born during 1925–1929, 12% were born during 1930–1934, 5% were born during 1935–1939, 5% were born during 1940–1944, and 8% were born during 1945–1950 (figure 1). A total of 128 men (60%) and 85 (40%) women were included.

The isolates demonstrated a highly homogenous population with little genetic variation although identical isolates were not included. The T- (40%) and Haarlem- (35%) families dominated among the isolates and only 25% belonged to other families (table 1).

**Table 1:** *M. tuberculosis* families identified in cases of reactivated disease among patients born in Norway before 1950.

T families (40%)	Subfamily.	Number of isolates	% of total (214)
	T1	75	35%
	T2	2	0,93%
	T3	3	1,4%
	T4	5	2,3%
Haarlem families (35%)	Haarlem 1	33	15,4%
	Haarlem 3	41	19,1%
Other families (25%)	Beijing family	5	2,3%
	EAI1 family	3	1,4%
	EAI2 family	5	2,3%
	EAI4 family	2	0,93%
	33 family	3	1,4%
	34 family	3	1,4%
	35 family	1	0,46%
	36 family	5	2,3%
	X3 family	3	1,4%
	LAM1 family	5	2,3%
	LAM6 family	1	0,46%
	LAM8 family	4	1,8%
	LAM9 family	12	5,6%
	CAS family	1	0,46%
	S family	1	0,46%

**Figure 2:**
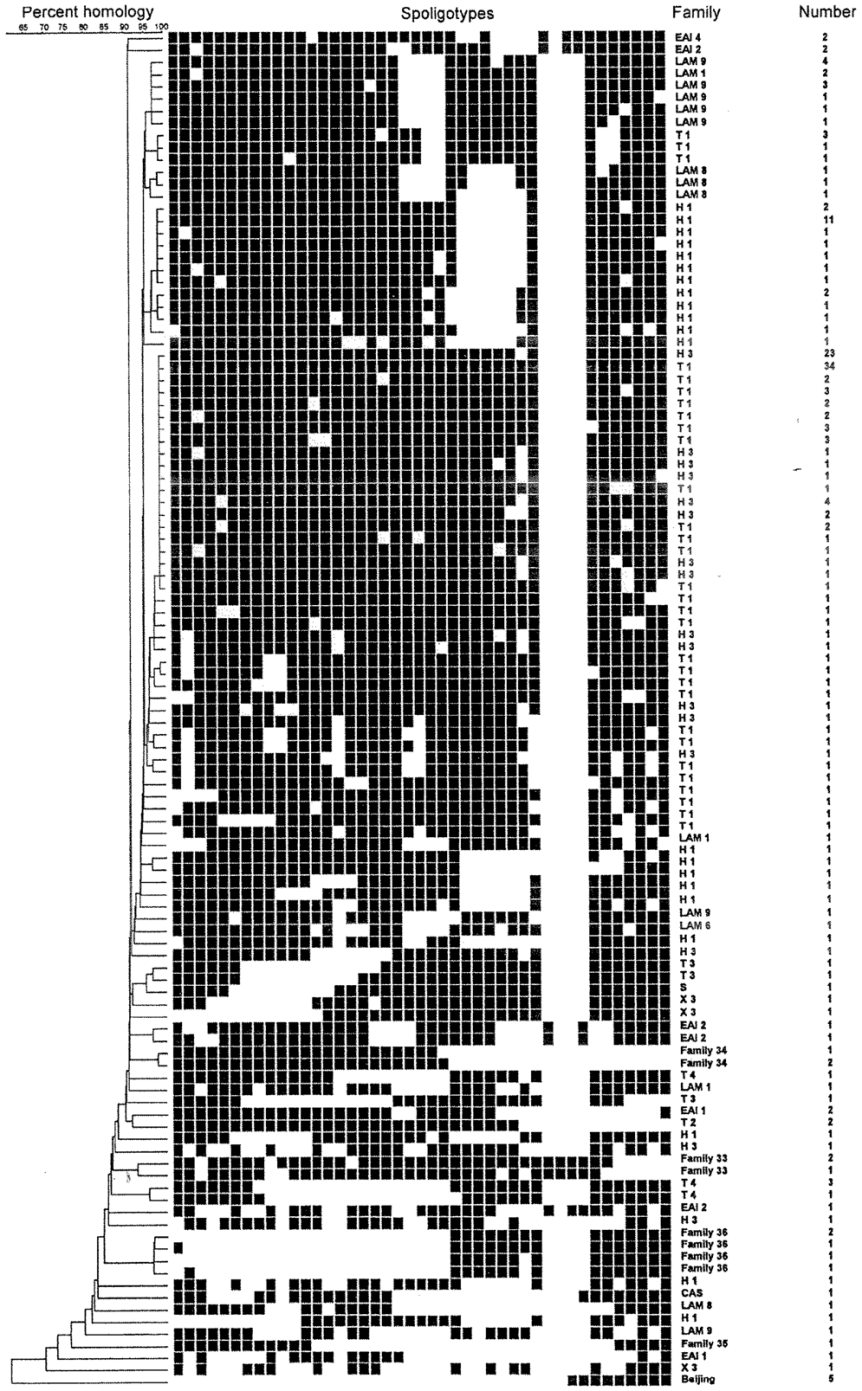
The diversity of spoligopatterns in *M. tuberculosis* isolates from patients born in Norway between 1910 and 1950. Percent homology, spoligopatterns, family assignment, and number of isolates carrying each pattern, among *M. tuberculosis* strains isolated from Norwegian-born patients during 1998–2008. All cases were considered reactivation of remote infection. Abbreviations: EAI: East African Indian-family, LAM: Latin American Mediterranean-family, T: T-family, H: Haarlem-family, S: S-family, X: X-family, CAS: Central Asian-family [7].

**Figure 3:**
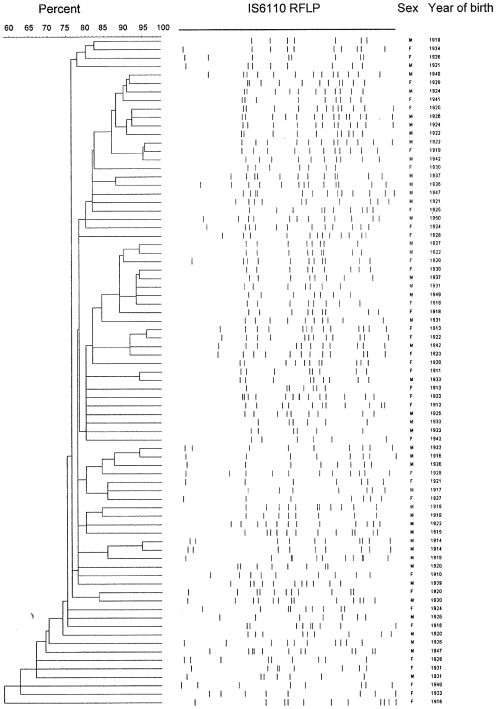
Diversity and RFLP patterns observed among *M. tuberculosis* strains, sex, and year of birth of patients from whom these were isolated. Genetic diversity and IS*6110*- RFLP patterns of the isolates assigned to the T- family from patients born in Norway 1910-1950.

**Figure 4:**
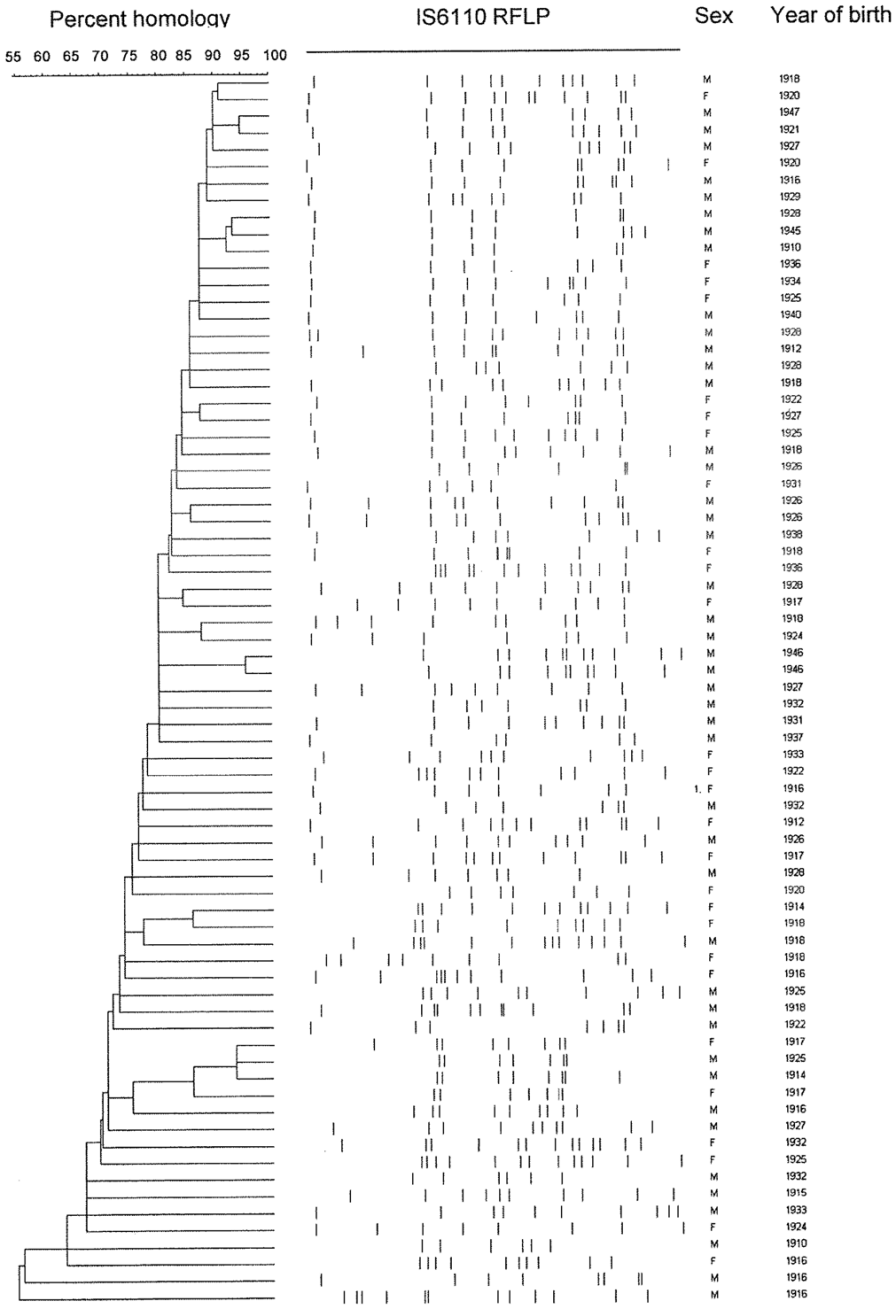
Diversity and RFLP patterns observed among *M. tuberculosis* strains, sex, and year of birth of patients from whom these were isolated. Genetic diversity and IS*6110*- RFLP patterns of the isolates assigned to the Haarlem- family from patients born in Norway 1910–1950.

Among the strains assigned to the Haarlem 1 sub-family, 24 carried identical spoligopatterns. All these were separated by IS*6110* RFLP yet the identical spoligopattern supported the hypothesis that the strains had a common evolutionary and epidemiological background (Table 1, Fig 2). Figures 3 and 4 demonstrates the genetic similarity among these strains obtained by IS*6110* RFLP analysis. All isolates are within a 70% homology and all single isolates demonstrate at least one very closely related strain although no clustering could be observed according to age, sex or current residence (data not shown). The IS*6110* RFLP dendrograms of the two dominant lineages were considered comparable to those of well established epidemics and represent local evolution rather that recent import and transmission.

A total of 23 among 41 (56%) isolates assigned to the Haarlem 3 family had identical spoligopatterns (Fig 2) and 21 of them were separated by IS6110-RFLP although their genetic similarity was above 70%. The T1 family demonstrated similar diversities as that observed among the Haarlem 1 and Haarlem 3 isolates. Among the 214 isolates analyzed in this study, were 75 identified as T1 family by spoligotyping and 51.9% of the isolates within this family had identical spoligopattern. The IS6110 RFLP analysis results differentiated the isolates and demonstrated a similarity above 65%.

## Discussion

The *M. tuberculosis* strains included in the current study were isolated in patients born in Norway between 1910 and 1950. Norway is a relatively small country with a currently low incidence of *M. tuberculosis* infections. Among 300–400 notified cases each year, 200–300 are culture positive. Since 1994, all cultures of M. tuberculosis are analyzed with IS6110 RFLP at the Norwegian Institute of Public Health [5]. The DNA patterns of the included strains had not previously been observed in the IS*6110* RFLP database, and where therefore unlikely to have been recently transmitted in Norway after 1993. Since travel-records of the patients were incomplete, it could not be ruled out that some of the patients were recently infected outside Norway. However, the low rate of diversity in the *M. tuberculosis* population strongly indicated that the majority of these isolates had a common epidemiological background and represented the remaining cases of the tuberculosis epidemic that plagued Norway prior to the 1960s [9]. During the first half of the 20^th^ century, Norway experienced large regional differences of tuberculosis infection and mortality. The northern part of the country had the highest infection rates and mortality due to tuberculosis [9]. Such differences were not observed in the current analysis, as the genotypes of *M. tuberculosis* were evenly distributed throughout the country. Internal migration within Norway is low and migration between different parts of the country remains stable at 5–7%. The majority of internal migrants are in the age group 20–34 years (statistics Norway: www.ssb.no). Thus the patients of the current analysis represented a group that rarely migrated within Norway.

Spoligopatterns of *M. tuberculosis* evolve through the successive loss of DNA spacers which separate the short direct repeat sequences in the *M. tuberculosis* genome. Deletions of such spacers need not be independent events and identical patterns may develop in unrelated strains as a result of convergent evolution [10], [11]. The method has however been in use for more than a decade and tens of thousands of strains have been typed to describe the various lineages of *M. tuberculosis* that dominate in various parts of the world. These studies have resulted in the detection and description of emerging strains that may represent vaccine escape variants or strains that acquire drug resistance more readily than others, or harbor other characteristics that render them especially successful [12], [13].

When tubercle bacilli first enter human bodies they usually remain through the rest of their hosts' lives and are capable of causing clinical disease any time. Tuberculosis has thus long been prevalent among elderly people. In the current study the Haarlem and T lineages as defined by spoligotyping [7], dominated among Norwegian-born patients diagnosed with tuberculosis that were considered not recently transmitted in Norway. The T1 and Haarlem 3 sublineages were the most commonly observed spoligopatterns. The Haarlem 3 pattern was also observed in 18^th^ century Hungarian bodies [14], demonstrating that this particular lineage had been present in Europe for centuries. It has been demonstrated that *M. tuberculosis* strains isolated in Denmark during the 1960s were carried as latent infections during 4 decades, without changing their IS*6110* RFLP patterns [15]. The Danish strains also carried IS*6110* RFLP patterns of high homology to those included in the current study. The Danish comparison between historical and recently isolated strains of *M. tuberculosis* yielded convincing evidence that reactivation occurred many decades after initial infection [15]. In Hong Kong, old age home residents have a high prevalence of latent tuberculosis that is responsible for the high rate of active disease due to reactivation. There it was concluded that elderly represents a large reservoir of tuberculosis infection [16], [17]. For these reasons, we believe that the Norwegian century-old tuberculosis epidemic, which is currently in the elimination phase, was partly caused by *M. tuberculosis* isolates within the T and Haarlem families.

Numerous projects have identified *M. tuberculosis* strains that may represent emerging types and that harbor higher abilities to resist anti-TB drugs and/or BCG vaccination [18]–[22]. Vaccines that target only a proportion of a specific pathogen population may exert selective pressures that can promote vaccine escape variants or even promote changes in microbial virulence [23], [24]. The efficacy of new tuberculosis vaccines and drugs, risk-factors for infections and other epidemiological aspects need to be correlated to emerging strains [23]. However, the strains that are identified as emerging are not simply those with the largest number of cases [20].

The lineages of *M. tuberculosis* that dominated among elderly patients diagnosed with assumed reactivated tuberculosis during 1998–2008, probably represented strains that are about to be eliminated after more than a century of tuberculosis control efforts in Norway [9]. It is not known how long each patient had been latently infected. However, the transmission rate of *M. tuberculosis* in Norway is currently very low [5], and the close genetic relatedness between the isolates indicated that they were acquired from a local epidemic in the past.

Strains of *M. tuberculosis* that were transmitted in the past and developed disease after long periods of latent infection, need not be a representative part of the bacterial population that circulated previously. Since various strains of *M. tuberculosis* harbor different levels of pathogenicity, levels of drug resistance, or other phenotypic characteristics [21], [25], it is possible that specific strains that demonstrated short latency-periods, or high mortality-rates in patients have already been eliminated from the Norwegian-born population. Such strain related differences have been recently demonstrated in other studies [26,26] However, we believe that the Haarlem 1, Haarlem 3 and T 1 families represent parts of the TB epidemic that scourged Norway during the first half of the 20th century. These strains are still abundant in many parts of the world [7], but should not be considered emerging as they represent strains that circulated in a high-incidence setting several decades ago.

A general goal in the control of infectious diseases has been to classify isolates into distinct epidemiological, immunological or virulent types. In the case of *M. tuberculosis*, one of the outcomes of genetic typing has been the identification of particularly aggressive or successful strains. The T and Haarlem strains are usually not considered among such successful strains, although they remain highly endemic in most parts of the world [7]. They have been assigned to the “modern *M. tuberculosis* strains” [23], [27], [28], and appear to have been successfully controlled in Norway through a rigorous control program described elsewhere [5]. Clear definitions of the “T” families (modern tuberculosis strains) are still absent and more than 600 unclassified spoligopatterns have been found among these strains [7]. The T and Haarlem strains that were abundant in the old-age Norwegian population studied in the current paper has been repeatedly isolated in the countries of the former Soviet Union [7], [29]. They are generally not associated with drug resistance on major outbreaks, thus epidemiologically the current isolates share these phenotypic traits.

The T 1, Haarlem 1, and Haarlem 3 families of *M. tuberculosis* were abundant among patients born in Norway between 1910 and 1950. The *M. tuberculosis* cases represented reactivated disease that had been acquired before 1994 and were likely to have been latent for several decades. Thus, the current study indicated that the T 1, Haarlem 1, and Haarlem 3 families were common in Norway, when tuberculosis represented a serious public health threat during the first half of the 20^th^ century.
